# Correlation of Leptin With Acute Myocardial Infarction: A Case Control Study

**DOI:** 10.7759/cureus.12190

**Published:** 2020-12-20

**Authors:** Asghar Hussain Syed, Sameer Lohana, Norah H Aung, Muhammad Khizar Memon, Anam Shaikh, Sidra Memon, Syeda M Hassan, Besham Kumar

**Affiliations:** 1 Internal Medicine, Royal College of Physicians, London, GBR; 2 Cardiology, National Institute of Cardiovascular Diseases, Karachi, PAK; 3 Family Medicine, Ziauddin University, Karachi, PAK; 4 Medicine, Liaquat University of Medical and Health Sciences, Karachi, PAK; 5 Health Sciences, Western Illinois University, Macomb, Illinois, USA; 6 Internal Medicine, University of Medicine (1), Yangon, MMR; 7 Internal Medicine, Liaquat University of Medical and Health Sciences, Hyderabad, PAK; 8 Internal Medicine, Dow University of Health Sciences, Karachi, PAK; 9 Internal Medicine, Jinnah Sindh Medical University, Karachi, PAK; 10 Medicine, Jinnah Sindh Medical University, Karachi, PAK; 11 Internal Medicine, Jinnah Postgraduate Medical Centre, Karachi, PAK

**Keywords:** leptin, acute myocardial infarction, pakistan

## Abstract

Introduction: Leptin, a satiety hormone, has the ability to inhibit hunger and is thus, a regulator of body weight. Leptin is also elevated in cardiovascular events such as acute myocardial infarction (AMI) and hence is considered as modifiable risk factors for AMI. The purpose of this study is therefore to investigate the correlation of leptin with AMI.

Methodology: In this retrospective study, data of patients were taken from the database between January 2017 to December 2019 from a cardiovascular unit of tertiary care hospital in Pakistan. Patients were divided into two groups, based on participants who had suffered from AMI and other groups who had not suffered an AMI. Leptin levels were compared for both groups. Age, body mass index (BMI), and smoking history were noted in a self-structured questionnaire. In addition, mean blood pressure and cholesterol levels were also noted for both groups.

Results: Leptin was significantly higher in patients with first time AMI (29.21 ± 9.21 ng/mL vs. 11.23 ± 3.12 ng/mL: p-value, <0.0001). BMI (27.2 ± 3.2 kg/m^2^ vs. 24.9 ± 2.8 kg/m^2^: p-value, <0.0001) and percentage of smokers (40.9% vs. 22.9%: p-value, 0.032) were also significantly higher in patients with AMI.

Conclusion: A positive correlation was found between AMI and serum leptin levels in smokers and obese patients. Hence, we suggest that cardiologists should stress upon controlling these modifiable risk factors to reduce the incidence of AMI in the future.

## Introduction

Leptin is a polypeptide hormone secreted by adipocytes and circulates through the bloodstream to exert its effects on the hypothalamus regulating body weight and energy balance. Leptin levels are decreased during fasting and are increased after feeding to inhibit appetite, making it an anti-obesity hormone [1,2]. Paradoxically, high levels of leptin have been strongly associated with high body fat, suggesting resistance to its effects on target organs [1]. This was confirmed by studies that showed overexpression of the ob gene in adipose tissues, mutation of which leads to profound obesity and type 2 diabetes [3]. Other than obesity, one study linked chronic hyperleptinemia with increased insulin resistance and diabetes mellitus type 2 (DM2), which further decreased pancreatitis beta-cell responsiveness creating a positive feedback loop [4]. Apart from that, multiple studies have reported the role of leptin in regulating myocardial blood flow, angiogenesis, arterial thrombosis and inflammatory vascular response [1,5,6]. Both obesity and DM2 are major modifiable risk factors for acute myocardial infarction (AMI) which is defined as the most severe form of acute coronary syndrome (ACS), diagnosed by rising levels of biochemical markers (such as troponin T and CK MB) of myocardial necrosis, ST-segment elevation on ECG and signs/symptoms of ischemia [7]. Other modifiable risk factors include increased triglycerides, low levels of high-density lipoprotein, systemic hypertension, and all five are part of metabolic syndrome [8].

Pakistan has an estimated prevalence of metabolic syndrome ranging from 18% to 46% [9]. One systemic analysis evaluated a 2% increase in the risk of AMI, cardiovascular disease (CVD) and CVD-related mortality in patients diagnosed with metabolic syndrome [8]. Various studies have proposed leptin as a predictive marker for metabolic syndrome [10]. Significant correlation of leptin levels with metabolic syndrome and thus as a biomarker for increased risk of AMI has not been studied in Pakistan yet. This article focuses on how leptin levels can directly lead to an increased risk of developing AMI among the Pakistani population.

## Materials and methods

This retrospective study was conducted in a tertiary care hospital in Pakistan. Data were taken from the cardiovascular patient database from January 2017 to December 2019. Patients who had a minimum of three values of leptin were included in this study. The patient who had a previous history of AMI were excluded from this study. Based on the inclusion criteria, 61 patients with AMI were enrolled in this study. Sixty-one participants from the cardiovascular patient database without AMI were included in the study as a referent (control group). This study was conducted in accordance with the declaration of Helsinki and ethical review board approval was taken.

Patients who fulfilled the inclusion criteria, their demographics such as age, body mass index (BMI), and smoking history were noted in the self-structured questionnaire. Mean blood pressure, cholesterol and leptin levels were noted for both groups. 

Statistical analysis was done using SPSS v. 22.0 (IBM Corporation, Armonk, NY). Continuous variables including age, blood pressure, BMI, cholesterol levels and leptin levels were analyzed via descriptive statistics and were presented as means and standard deviations (SDs) while categorical variables such as smoking history were presented as percentages and frequencies. T-test and Chi-square were applied as appropriate. A P-value of less than 0.05 meant that differences between the groups are significant and the null hypothesis is void.

## Results

Leptin was significantly higher in patients with first time AMI (29.21 ± 9.21 vs. 11.23 ± 3.12: p-value, <0.0001). BMI (27.2 ± 3.2 vs. 24.9 ± 2.8: p-value, <0.0001) and percentage of smokers (40.9% vs. 22.9%: p-value, 0.032) was also significantly higher in patients with AMI. There was no significant difference in systolic blood pressure, diastolic blood pressure and cholesterol levels (Table 1).

**Table 1:** Comparison of parameters between case and control groups AMI: acute myocardial infarction, SD: standard deviation, BMI: body mass index.

Characteristics (mean ± SD)	Patients with AMI (n=61)	Patients without AMI (n=61)	P-value
Age (years)	51 ± 11	50 ± 11	0.61
Systolic blood pressure (mm Hg)	141.2 ± 16.2	137.1 ± 14.5	0.14
Diastolic blood pressure (mm Hg)	89.2 ± 11.0	86.1 ± 9.2	0.09
Cholesterol levels (mg/L)	192.2 ± 31.9	186.3 ± 27.2	0.27
Smoking status (%)	25 (40.9%)	14 (22.9%)	0.032
BMI (kg/m^2^)	27.2 ± 3.2	24.9 ± 2.8	<0.0001
Leptin (ng/mL)	29.21 ± 9.21	11.23 ± 3.12	<0.0001

## Discussion

Recently, multiple studies have proved the association of increased levels of leptin with AMI, although insufficient data are available to list leptin as an independent factor for the risk of AMI [11]. Other cardiovascular diseases where high serum leptin level is reported to be associated with risk are stroke, left cardiac hypertrophy and chronic heart failure [12]. In our study, we analyzed levels of leptin, BMI, smoking status, diastolic and systolic blood pressure and cholesterol levels in patients with AMI versus patients without AMI.

In the present study, AMI was found mostly in the middle-aged group. This result corresponds to a study in Japan where there is an increasing trend of AMI in population ≤59 age with a decreasing incidence in elderly group ≥70 age during the last decade [13]. We also found out significantly high levels of leptin in the acute AMI group [29.21 ± 9.21 (p = <0.0001)] than the non-AMI group (11.23 ± 3.12). In correlation with this, another comparative study (AMI vs control group), found out high levels of serum leptin, C-reactive protein, endothelin and troponin T levels in AMI patients. All except troponin was also found in patients with coronary atherosclerosis and were found to be independent of each other [14]. Morita et al. enrolled 724 patients with AMI who underwent successful emergency percutaneous coronary intervention (PCI) and checked their level of leptin and adiponectin after seven days of AMI. They found a higher level of leptin and a lower level of adiponectin with an increasing incidence of AMI. Moreover, with three-year follow-up, they observed 63 adverse effects, all relating to a higher ratio of leptin to adiponectin and concluded the importance of leptin to adiponectin ratio as an independent prognostic factor for AMI [15].

As we have already discussed the association of hyperleptinemia with AMI, the underlying mechanism is not fully understood [12]. One pathway has been thoroughly studied in regarding AMI as an inflammatory process. Tumor necrosis factor-alpha (TNF-α) is an important pro-inflammatory mediator and is linked with the risk of recurrent myocardial infarction and death [16,17]. Physiologically, leptin has a suppressive effect on the expression of TNF-α, however, evidence suggests that hyperleptinemia can trigger an inflammatory response by activating TNF-α via p38 and JNK (c-Jun N-terminal kinase) MAPK (mitogen-activated protein kinases) pathway in adipose tissues especially in obese individuals [12,18]. Also, studies have found the role of TNF-α in insulin resistance in obese patients, suggesting its involvement in DM2 [12]. Another possible mechanism can be understood by studying the role of leptin in atherogenesis. Leptin transmits a signal by binding to its receptor Ob-R, which is mainly found in the hypothalamus but is also expressed in macrophage, endothelial cell, and smooth muscle cells. Inflammation, dysfunction of endothelial cells and migration of smooth muscles to the injured intima are an important event in the formation of plaque, thus contributing to cardiovascular disease [19].

Interestingly, a research on 122 AMI patients admitted in ICU showed no notable change in serum leptin level but was elevated in patients with obesity, high BMI and waist circumference [20]. The author concluded that perhaps leptin level is independently associated with high BMI and obesity which is well known to be related with cardiovascular diseases [21].

In our study, smoking and a high level of BMI, both were significantly present in patients with AMI. Current smoking is associated with all types of cardiovascular diseases, with twice the risk for AMI. Moreover, quitting smoking before 45 years substantially reduces the risk [22]. On the other hand, it is already proven that high BMI as a part of metabolic syndrome possess a great threat for AMI [7,8]. Nevertheless, a study on 13,104 AMI patients in Korea found out that higher BMI (≥26 kg/m^2^) proved to a good prognostic factor post-one-year AMI in contrast to lower BMI (<22 kg/m^2^) [23].

Although modifiable risk factors for AMI include hypertension, hyperlipidemia, diabetes mellitus, obesity and smoking history [24], our study did not find any significant changes in blood pressure and cholesterol levels. The author believes that the disparity in results could be due to the small sample size and/or decreased age of enrolled patients. Our study indicates leptin levels might be a predictor for future myocardial infarction. Furthermore, large-scale studies are required to validate our findings.

## Conclusions

This retrospective study concludes that anti-obesity hormone namely, leptin spikes up in patients with the first occurrence of myocardial infarction which can be due to target organ resistance to leptin in such patients. The data from Pakistan are, however, limited but a correlation has also been found between hyperleptinemia and metabolic syndrome, which in turn leads to AMI and its morbid outcomes. More detailed and widespread research is required to establish a definite correlation between leptin levels and AMI.
